# Early Exposure to Respiratory Allergens by Placental Transfer and Breastfeeding

**DOI:** 10.1371/journal.pone.0139064

**Published:** 2015-09-23

**Authors:** Patricia Macchiaverni, Leandro H. Ynoue, Christina Arslanian, Valérie Verhasselt, Antonio Condino-Neto

**Affiliations:** 1 Department of Immunology, Institute of Biomedical Sciences, University of São Paulo, São Paulo, Brazil; 2 Institut National de la Santé et de la Recherche Médicale (INSERM), U924 Université de Nice-Sophia Antipolis, Valbonne, France; INSERMU1138, FRANCE

## Abstract

The relationship between allergen exposure and the onset of or protection from allergic diseases remains unclear. Many factors could be related to immunological responses, such as the age when the exposure occurs, type of allergen, timing, dose, and allergen route. In this study, we investigated whether exposure to respiratory allergens could occur in pregnancy or early life. In particular, we assessed whether Der p 1 and Blo t 5, as well as specific antibodies against these allergens, could be detected in 90 paired cord blood and colostrum samples. Der p 1 was detected in 58.6% of colostrum and 29% of cord blood samples, whereas Blot 5 was positive in 41.3% and 9.6% of the samples, respectively. Similar to specific IgA, which could be detected in all samples for both mites, specific IgG was found in a high number of colostrum samples, 93.5% and 94.8% for Dp and Bt, respectively. Although allergens were not detected in all cord blood samples, a high percentage of them (≥95%) were positive for specific IgM to both mites in cord blood samples, suggesting that neonates can be exposed and sensitized to airborne allergens during pregnancy. Many studies have attempted to correlate allergen exposure or its prevention in early infancy with the onset of or protection from allergic diseases. However, conflicting and inconsistent data do not show a clear correlation with or suggest a way to prevent allergen sensitization. Nevertheless, these unconvincing results could be better understood if the relationship with many aspects of allergen exposure after pregnancy could be clarified. Thus, it is necessary to address basic issues related to allergen exposure, including the development of reproducible, standardized and reliable methods, and to determine how and where the exposure occurs.

## Introduction

Allergic respiratory diseases are an important public health issue. They display a high rate of incidence and prevalence and represent a major burden. The high susceptibility of children to allergies is related to environmental factors, such as diet, air pollution, tobacco smoke, allergens, and stress [1]. The main factors responsible for of the majority of allergic diseases are environmental agents, particularly indoor allergens. The mite *Dermatophagoides pteronyssinus* (Dp) is the major allergen source in house dust and is one of the most frequently implicated in asthma [2]. Additionally, *Blomia tropicalis* (Bt) is very common in tropical countries and responsible for an equally large amount of sensitization in these regions [3].

Regarding allergen exposure, many studies on the importance of avoiding allergens to prevent sensitization and the development of asthma remain controversial and need to be clarified. An important factor is that the measurement of allergens that are present in the environment would not necessarily correspond to an individual’s allergen exposure. Another question is when and what type of allergen exposure occurs throughout life, and then if the subject will develop an allergic disease or tolerate the allergen. Moreover, the route of exposure is likely to be important. In contrast to the assumption that inhaled allergens enter through the respiratory tract, other routes of exposure include oral, breastfeeding, cord blood, and even trans-cutaneous, and the outcomes of these different types of allergen exposure remain unclear [4].

The transfer of IgG antibodies through the placenta is very important and essential to protect children during early life. After birth, the colostrum and breast milk confer protection once the child comes into contact with the environment. Several studies have analyzed specific IgE to airborne and food allergens in the cord blood and correlated them to exposure to these sources [5–11]. The pathogenesis of allergic disease is influenced by complex interactions between genetic components and environmental changes, including the amounts and timing of initial exposure to airborne allergens [12]. Placental transfer and breastfeeding are also potential routes of allergen exposure in very early life and might exert effects on newborns through still poorly defined immunological pathways [13]. In addition to allergen transfer, some epidemiologic evidence indicates that maternal antibodies can influence susceptibility to allergic disease in offspring [14].

Studies showing allergens and specific antibodies in the colostrum or breast milk are rare and generally related to food allergens [15–19]; therefore, in two studies by our group, we measured specific IgA and IgG to mite allergens [13, 20]. The presence of airborne allergens has been shown in amniotic fluid and cord blood samples by detecting specific allergens or immune complexes, and intrauterine sensitization has been confirmed along with the detection of specific IgE in cord blood samples [21–23]. Maternal influences on infant susceptibility to allergic disease remain poorly understood in humans, likely because we still do not know how aero-allergen exposure occurs in the first years of life. It is important to better understand all aspects of allergen exposure before we classify them as a protective or sensitizing factor. With this in mind, this study aimed to investigate the two main respiratory allergens for our geographic area [24–27] and whether specific antibodies could be transferred through trans-placental and breastfeeding routes.

## Methods

### Ethics Statement

The selected women who agreed to join the study were informed about the procedures and signed the informed consent. The study was approved by the Ethics Committee of the Institute of Biomedical Sciences, University of São Paulo, in accordance with the Brazilian Ministry of Health Resolution 96⁄1996 and the Helsinki Declaration.

### Study design

Women who gave birth to healthy term infants at Hospital Maternidade de Campinas in São Paulo, Brazil, were invited to join the study during their hospital stay. Exclusion criteria for enrollment were hypertension, diabetes, infections, immunodeficiency, and having received corticosteroids, blood transfusion, or other drugs related to chronic diseases during pregnancy.

### Samples

Cord blood was collected from large veins on the fetal side of the placenta that had been sterilized with ethanol immediately after delivery. Maternal blood was collected within 48 h after delivery, and both maternal and cord blood serum were separated and stored at –80°C until assayed. Colostrum samples were collected manually within 48 h after delivery and then centrifuged for 30 min at 160×*g* at 4°C. The top layer of fat and the cell pellet were discarded, and the intermediate fluid fraction was stored at –80°C until analyzed.

### Dp- and Bt specific IgE quantification

Specific IgE antibodies from maternal serum samples against Dp and Bt allergens were analyzed by chemiluminescent immunoassay (Cap System Pharmacia^®^) according to the manufacturer’s recommendations [28]. The specific IgE concentration is expressed in kU/L; values ≥0.35kU/L were considered to be positive for specific IgE. Because of their epidemiological relevance for our geographic area, we focused our study on these two indoor airborne allergens [24–27].

### Quantification of Dp- and Bt-specific antibodies

Dp- and Bt*-*specific total IgG, IgG1, IgG2, IgG3, and IgG4 were measured in paired newborn and maternal serum samples; specific IgM was measured in newborn serum, and specific IgG and IgA were measured in colostrum samples. All quantifications were performed using ELISA as previously described, with some modifications [13]. Microplates (Costar, Cambridge, MA, USA) were coated overnight at 4°C with 5 μg/mL Dp or Bt extract (both from Greer Laboratories, Lenoir, NC, USA) diluted in 100 μL phosphate-buffered saline (PBS) solution. Plates were saturated with 5% non-fat dry milk in PBS-Tween 0.1% for 1 h at room temperature. The samples were added in duplicate followed by two-fold serial dilution and by incubation with secondary antibodies; both were diluted in the same blocking buffer and incubated for 2 h at 37°C. Streptavidin-HRP (554066, BD Pharmingen) was used as a secondary reagent to detect biotinylated antibodies, and Ortho-phenylenediamine (OPD) was used as a chromogenic substrate. For Bt*-*specific IgG4 quantification in maternal and newborn serum, we performed an amplified ELISA using an ELISA amplification system (19.589–019, Invitrogen) according to the manufacturer’s instructions. Additionally, two quality-control samples and two negative samples (blank) were included on each plate. Data are expressed as arbitrary units (AU/ml) obtained by comparison with positive serum or a colostrum pool defined to contain 1000 AU/ml specific IgG or IgA. The sample dilutions and antibody concentrations are summarized in Table 1.

**Table 1:** Antibody and sample dilution for specific IgA, IgG and IgM antibodies against *B*. *tropicalis* and *D*. *pteronyssinus*, as measured by ELISA assays.

Sample	[dilution Dp/Bt]	Antibody [dilution DP/Bt]	
**Serum**			
IgG	[1:200/1:1000	anti-human IgG HRP (A8419; Sigma)	[1:400/1:400]
IgG 1	[1:5/1:2]	anti-human IgG1 biotin (555869; BD)	[1:500/1:500]
IgG 2	[1:2/1:2]	anti-human IgG2 biotin (555874; BD)	[1:100/1:500]
IgG 3	[1:4/1:4]	anti-human IgG3 biotin (B3523; Sigma)	[1:200/1:200]
IgG 4	[1:2/1:2]	anti-human IgG4 biotin (555882/555879; BD)	[1:100/1:200]
IgM	[1:4/1:4]	anti-human IgM biotin (B1265; Sigma)	[1:500/1:500]
**Colostrum**			
IgA	[1:100/1:50]	anti-human IgA HRP (A0295; Sigma)	[1:6000/1:6000]
IgG	1:2/1:2	anti-human IgG biotin (555785; BD)	[1:500/1:200]

### Allergen (Der p 1 and Blo t 5) quantification

Der p 1 and Blo t 5 were measured in the colostrum and cord blood serum using a Der p 1 ELlSA kit, 5H8/4Cl (EL-DP1-Indoor Biotechnologies), and a Blo t 5 ELISA kit (EL-BT5- Indoor Biotechnologies), with some modifications. Briefly, 96-well Maxi-Sorp plates (Nunc 442404) were coated overnight at 4°C with 2 μg/mL anti-Der p 1 mAb 5H8 or anti-Blo t 5 mAb 4G9, which were both in carbonate-bicarbonate buffer, pH 9.6. The next day, plates were blocked with 150 μL 1% BSA in PBS-Tween 0.05% for 1 h at room temperature. Samples were added and then diluted 2-fold in the same blocking buffer, followed by incubation overnight at 4°C or for 2 h at 37°C for the Der p 1 and Blo t 5 assays, respectively. Samples were incubated for 2 h at room temperature with biotinylated anti-Der p1 mAb 4C1 or anti-Blo t 5 mAb 4D9 followed by streptavidin-HRP (554066, BD Pharmingen) for 30 min. TMB (50-65-00—KPL) was used as a chromogenic substrate. Plates were washed in PBS-Tween 0.05% between each step. Absorbance was measured at 450 nm and quantified according to the Der p 1 and Blo t 5 standard curves (Universal allergen standard, Indoor Biotechnologies). The Der p 1 and Blo t 5 detection levels were 30 pg/mL and 125 pg/mL, respectively.

### Statistical analyses

Statistical analyses were performed using GraphPad Prism version 5.00 for Windows (GraphPad Software, San Diego, CA, USA). Dots represent individual data points, and the horizontal line represents the median of each group. A Mann–Whitney test for non-paired samples and the Wilcoxon signed rank test for paired samples were used to determine significant differences because the D’Agostino–Pearson normality test was not passed. The correlation coefficients for the antibody levels in maternal serum versus colostrum or cord blood were determined using Spearman’s tests. Two-tailed *p*-values are presented, and *p*-values <0.05 were considered significant; * *p*<0.05; ** *p*<0.01; *** *p*<0.001; **** *p*<0.0001.

## Results

### Demographic data

A total of 90 pregnant women were selected to join this study. Their age ranged between 15 and 40 years (mean 26.5 years). 42 were sensitized to Dp and 31 to Bt, while 29 were positive for both. The allergic women had moderate/severe persistent rhinitis and/or mild/moderate persistent asthma. None of the women presented with a clinical history of food allergy. The newborns were 51% female and 49% male. Other characteristics of the study population, such as newborn weight and height, maternal gestation number and route of newborn delivery, are summarized in Table 2. The high percentage of Caesarean section births in Brazil is related to cultural, social, and demographic factors, as well as the Brazilian health care system [29]. All newborns included were born between gestational 38 and 42 weeks and therefore were classified as full term.

**Table 2:** Mother and child data.

Study population (n = 90)	Mean [range]
Maternal age	26.5 [15–40] years
Newborn weight	3.3 [2.3–4.5] kg
Newborn height	48.8 [44.5–53] cm
Newborn sex	49% male
	51% female
Route of delivery	23% vaginal
	66% caesarean
	2% forceps
Gestation number	39% first
	41% second
	20% ≥third
Sensitized mother’s ICS≥3*	
Dp	69%
Bt	42%

* ImmunoCap score (ICS—**0** <0.35kU/L; **1** 0.35–0.7kU/L; **2** 0.7–3.5kU/L; **3** 3.5–17.5kU/L; **4** 17.5–50.0kU/L; **5** 50.0–100.0kU/L; **6** >100.0kU/L).

### Cord blood

**Fig 1:**
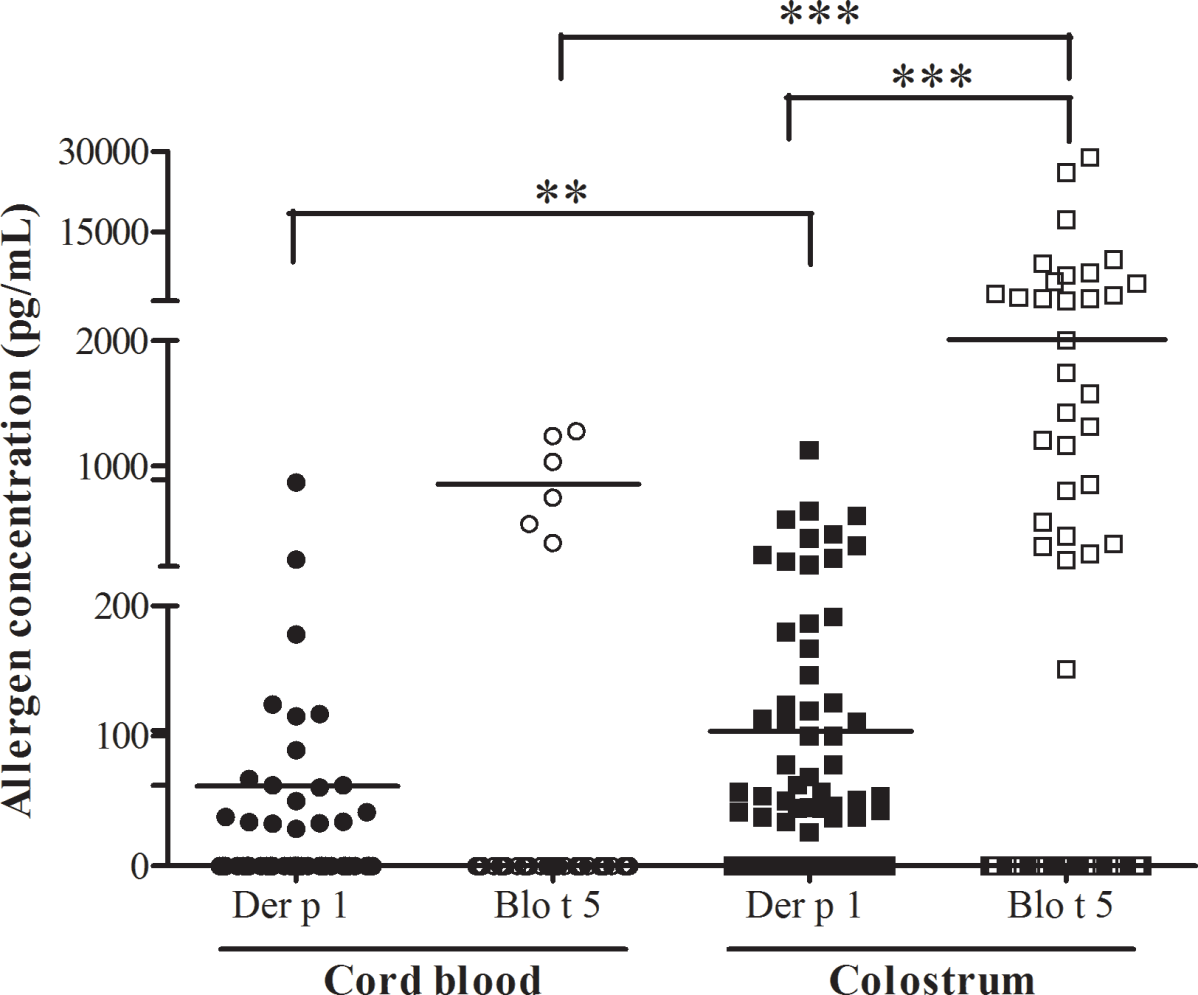
Levels of Der p 1 and Blo t 5 allergens in the cord blood and colostrum samples. Horizontal lines represent the median detected allergen levels. All *p*-values were determined using the Wilcoxon signed rank test; ** *p* < 0.01; ***p < 0.001.

The main allergens of *Dermatophagoides pteronyssinus* (Der p 1) and *Blomia tropicalis* (Blo t 5) mites were quantified in cord blood samples to assess the placental transfer of airborne allergens. We detected Der p 1 in 29% of cord blood samples, whereas Blo t 5 was detectable in only 9.6% of cord blood samples. Despite the low rate of positivity, levels of Blo t 5 were higher than those of Der p 1 (Table 3; Fig 1). No correlation was observed for IgG or any subclasses compared with their respective allergen levels in the cord blood samples (data not shown). Only three samples were quantified for both allergens, so no correlation analysis was carried out.

**Table 3:** Blo t 5 and Der p 1 in cord blood and colostrum samples.

Allergen (pg/mL)	Cord blood		Colostrum	
	Der p 1	Blo t 5	Der p 1	Blo t 5
Number of samples	62	62	75	75
Positive samples [%]	18 [29%]	6 [9.6%]	44 [58.6%]	31 [41.3%]
Concentration range	0–869.9	0–1281	0–1126	0–28,890
Median*	62.2	892.4	104.3	2000
25% percentile*	36.9	503.3	104.3	801.3
75% percentile*	118.8	1250	207.9	5667

* For only positive samples. Detection limit, 30 pg/mL for Der p 1 and 125 pg/mL for Blo t 5.

**Fig 2:**
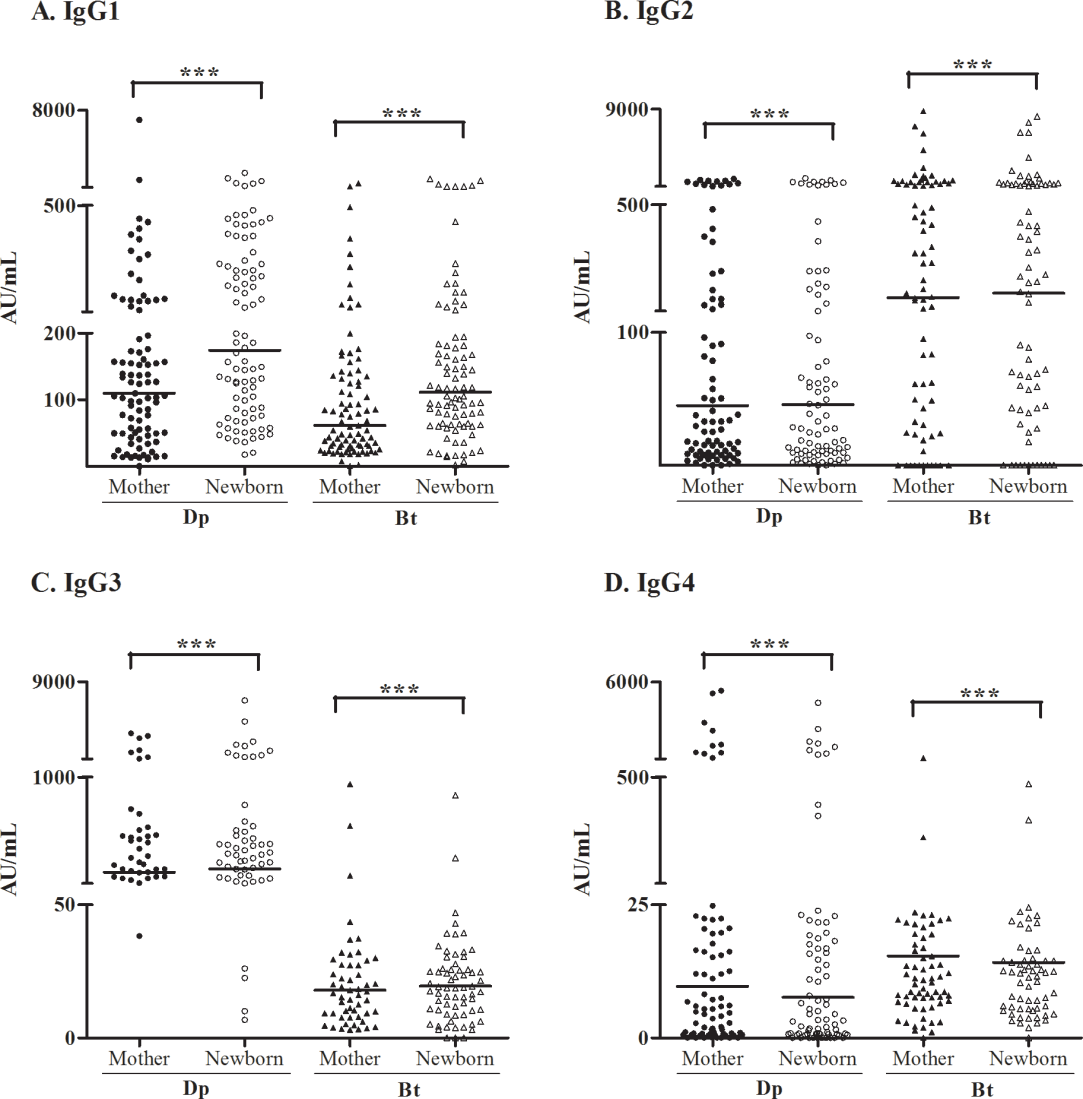
Levels of specific IgG subclasses to *D*. *pteronyssinus* (Dp) and *B*. *tropicalis* (Bt) in both cord blood and mother blood sera. Horizontal lines represent the median antibody levels; ***p < 0.001, as determined using the Mann–Whitney test.

Dp- and Bt-specific IgG1, IgG2, IgG3, and IgG4 were present in the cord blood of almost all neonates over a wide range of concentrations (Table 4). All IgG subclass antibody concentrations in the cord blood were strongly correlated with those in the maternal serum for both specificities (S1 Fig). Levels of IgG2 for Bt were higher than those for Dp, whereas the levels of IgG3 were higher for Dp than for Bt (Fig 2B and 2C). As we observed previously [13], levels of specific IgG were higher in atopic than non-atopic mothers (data not shown).

**Table 4:** Specific IgG subclasses to *B*. *tropicalis* and *D*. *pteronyssinus* in maternal serum and cord blood samples.

Specific-IgG in serum samples (AU/mL)	IgG1		IgG2		IgG3		IgG4	
	MB	CB	MB	CB	MB	CB	MB	CB
**Bt**								
Number of samples	87	88	84	84	57	73	89	85
Positive samples [%]	86 [98.8%]	88 [100%]	73 [89.9%]	72 [85.7%]	57 [100%]	70 [95.8%]	88 [98.8%]	84 [98.8%]
Concentration range	0–908	2.4–1324	0–9127	0–8226	3–947.7	0–861.7	0–567.6	0–481.5
Median*	64.3	111.1	336	344.4	17.9	20.9	15.8	14.3
25% Percentile*	30.5	62.8	112.3	81.8	9.2	11.5	8.0	7.1
75% Percentile*	136.6	183.1	933.2	800.7	29.7	32.3	34.3	35.1
**Dp**								
Number of samples	89	88	90	90	57	77	90	90
Positive samples [%]	88 [98.8%]	88 [100%]	87 [96.6%]	88 [97.7%]	57 [100%]	77 [100%]	82 [91%]	80 [88.8%]
Concentration range	0–7058	17–1902	0–1344	0–1393	38–3654	6.7–7059	0–5357	0–4498
Median*	109.6	174.1	45	45.7	287.4	309.4	9.6	7.6
25% Percentile*	49.3	81.6	10.8	10.6	172.1	147	1.1	0.8
75% Percentile*	200.8	335.5	217.5	195.2	551.3	509.4	26.7	25.2

* For only positive samples. MB, Maternal blood; CB, Cord blood. Detection limit, 1 AU/mL.

We also observed that >90% of samples were positive for specific IgM directed to Dp or to Bt total mite extract (Table 5). The levels of specific IgM for Dp correlated with Der p 1 levels in the cord blood (r = 0.52; *p* < 0.05), but no correlation was observed for specific IgM to Bt and Blo t 5 (S2 Fig).

**Table 5:** Specific IgM to *B*. *tropicalis* and *D*. *pteronyssinus* in cord blood samples.

IgM-specific in cord blood (AU/mL)	Dp-IgM	Bt-IgM
Number of samples	88	88
Positive samples [%]	84 [95%]	87 [98.8%]
Concentration range	0–11.7	0–83.4
Median*	3	2.3
25% percentile*	2.3	1.5
75% percentile*	4.2	3.6

* For only positive samples. Detection limit, 1 AU/mL.

### Colostrum

**Fig 3:**
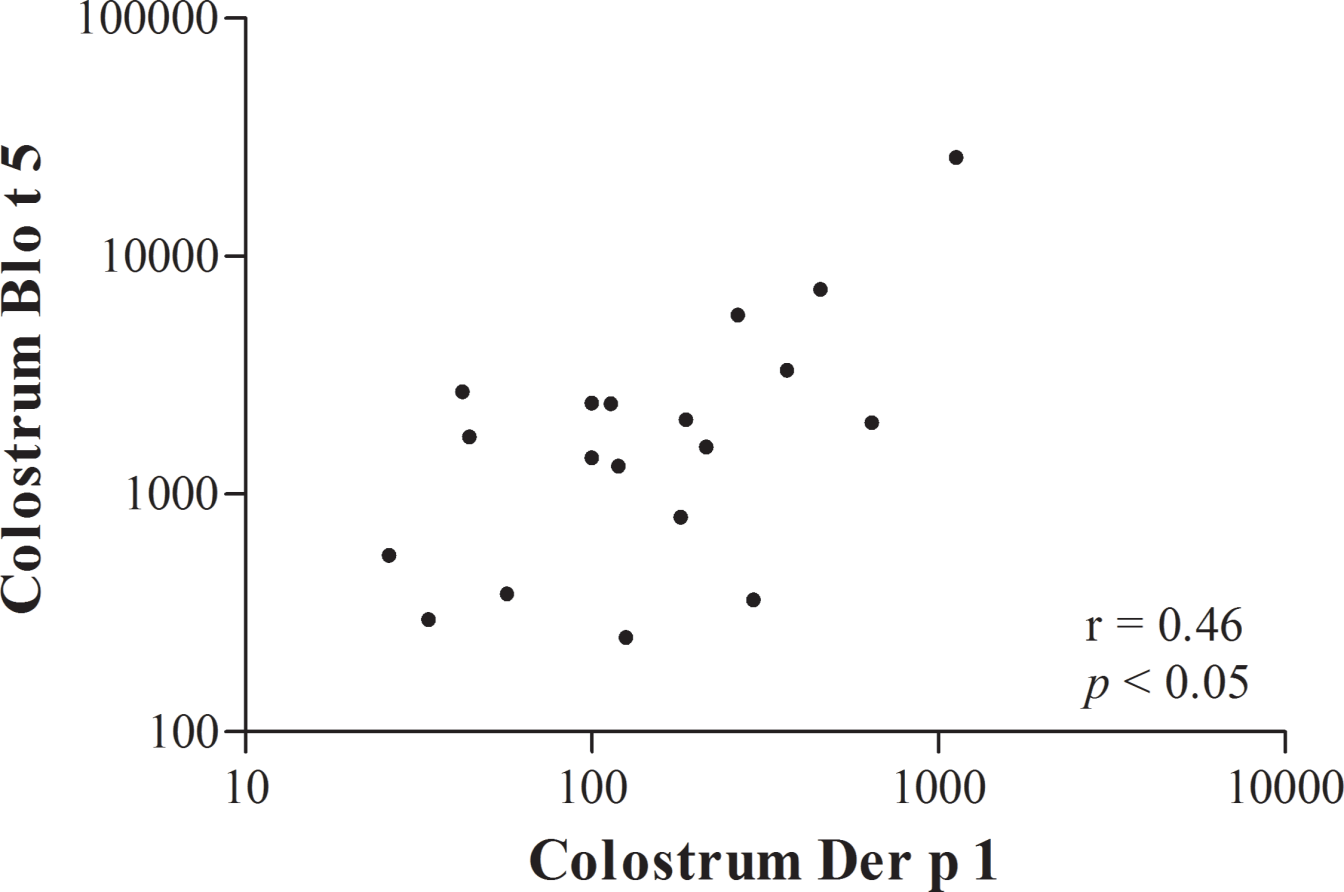
Correlations between Der p 1 and Blo t 5 in colostrum samples. Correlation coefficients were determined using Spearman’s tests.

The presence of Der p 1 and Blo t 5 HDM allergens in the colostrum samples were also analyzed to assess the transfer of airborne allergens through breastfeeding. Among those samples, 58.6% were positive for Der p 1 and 41.3% were positive for Blo t 5, in concentration ranges of 0–1126 pg/mL and 0–28,890 pg/ml, respectively (Table 3). Despite the huge variation in allergen concentration among mothers, median Blo t 5 concentrations in the colostrum samples were higher than that of Der p 1 (Fig 1). The Der p 1 and Blo t 5 levels were weakly correlated in the colostrum samples (Fig 3; Spearman test, r = 0.46, p < 0.05).

**Fig 4:**
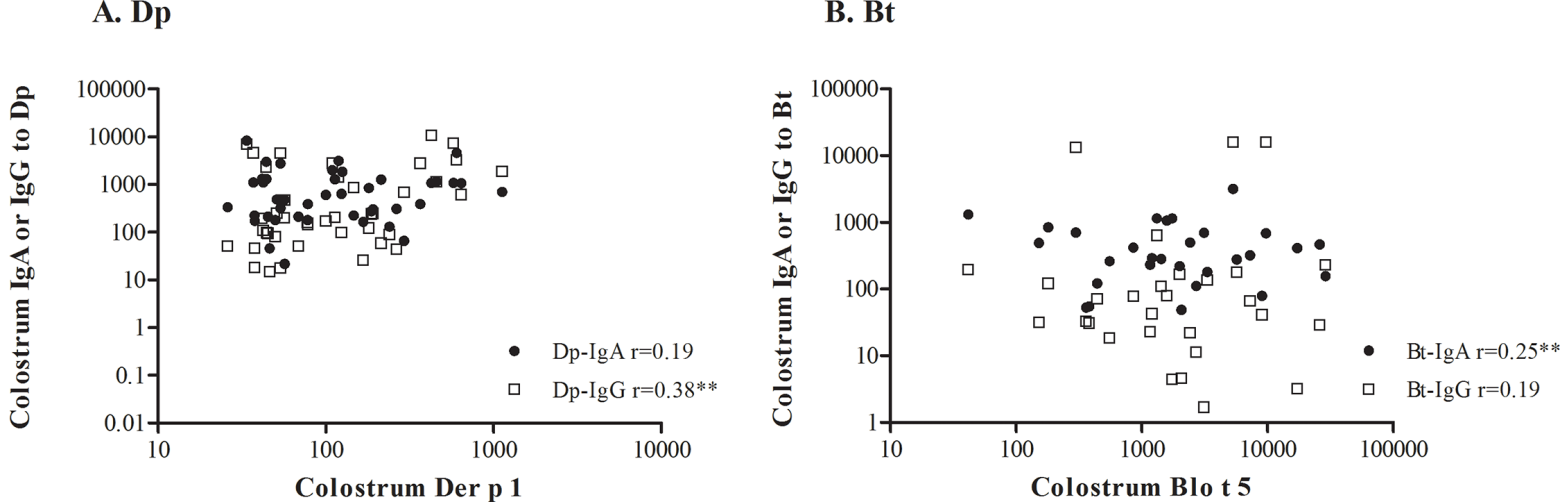
Correlation between specific IgA or IgG to Dp (A) or Bt (B) and Der p 1 (A) or Blo t 5 (B) allergens. Correlation coefficients were determined using Spearman’s tests; ** *p* < 0.01.

Specific IgA and IgG were measured over a wide range of concentrations for both Dp and Bt. Specific IgA was detected in all samples, and specific IgG levels were detected in a high percentage of the colostrum samples: 93.5% and 94.8% for Dp and Bt, respectively (Table 6). The concentration of Der p 1 in the colostrum was weakly correlated with Dp IgG levels, but not with colostrum Dp IgA levels. By contrast, Blo t 5 concentrations were weakly correlated to Bt-IgA levels, but not with Bt-IgG colostrum levels (Fig 4).

**Table 6:** Specific IgA and IgG to *B*. *tropicalis* and *D*. *pteronyssinus* in colostrum samples.

Specific antibodies in colostrum (AU/mL)	Dp		Bt	
	IgA	IgG	IgA	IgG
Number of samples	81	77	82	77
Positive samples [%]	81 [100%]	71 [93.5%]	82 [100%]	73 [94.8%]
Concentration range	21.5–8240	0–10,807	8.7–3178	0–19,060
Median*	463.3	198.8	250.4	31.7
25% percentile*	225.0	52.6	98.6	13.1
75% percentile*	1108	863.9	497.2	171.6

* For only positive colostrum samples. Detection limit, 1 AU/mL.

## Discussion

In this study, we demonstrated the presence of major respiratory allergens (Der p 1 and Blo t 5) in paired colostrum and cord blood samples. The detection of respiratory allergens in these samples shows that exposure to these allergens may occur very early in life, and correlations with the development of respiratory allergies could be studied in the future.

The relationship between exposure, sensitization and allergic manifestations is complex and will require additional study to be fully elucidated. There is no effective way to express all environmental factors (type of allergen, timing, pattern, dose or route of exposure) and compare them with the development of sensitization or allergic diseases [4]. Once sensitized to an allergen, the classical recommendation to prevent allergic manifestations is allergen avoidance. However, trials that assess at indoor environmental control aimed at reducing the risk of sensitization itself have produced no convincing results [30]. These unconvincing results could be related to the hygiene hypothesis [31], which holds that house dust has many other components and that some of them, such as microbial products, could be protective, as has been observed in some studies with endotoxins [32–34]. Furthermore, another important factor that requires better characterization is the interaction between genetics and environmental routes of exposure, as well as the route of allergen exposure to the immune system.

Herein, we demonstrate that although only a low percentage of newborns had detectable levels of Der p 1 (29%) and Blo t 5 (9.6%) in the cord blood, more than 90% of samples were positive for specific IgM, directed either to Dp or Bt total mite extract (Table 5). We know that among all five classes of human antibodies, only IgG is transferred across the placenta [35]. We detected Dp- and Bt-specific IgM in a large number of cord blood samples, demonstrating that neonates possibly had contact and became sensitized to these allergens, although polyreactive IgM natural antibodies may also influence these results [36]. Nevertheless, we were able to correlate specific IgM to Dp and Der p 1 in the cord blood, unlike for Bt and Blo t 5, most likely because of the low percentage of Blo t 5 detected in samples that resulted from the detection limit of the assay. Additionally, it has been demonstrated that Der p 1 could be detected in the amniotic fluid [21, 37], so this sensitization is not just related to the presence of allergens in our cord blood samples. Despite some studies that suggest the importance of early exposure in the development of allergies [38–42], the consequences of intrauterine exposure to allergens remain unknown, and there are no available data about the optimal age of exposure or even if there are differences related to when in life the exposure occurs.

In a study that detected ovalbumin (OVA) in breast milk and cord blood [43], a positivity rate of 35% was found in both samples (0.12–1258 ng/mL and 0.05–5.67 ng/mL, respectively). In colostrum samples, the positivity for Der p 1 and Blo t 5 was somewhat higher (58.6% and 41.3%, respectively); however, they collected breast milk 3 months after the birth, while we collected it after 2 days. This variation in concentrations could be related to the time of sample collection or even to the different routes of exposure to allergen sources. Whereas in cord blood samples the positivity for Der p 1 (29%) was similar, it differed for Blo t 5 (9.6%), probably because of the detection limit of our assay, which was 30 pg/mL and 125 pg/mL, respectively.

The transplacental passage of allergens has been demonstrated in previous studies and indicates the existence of an active transport mechanism because allergen levels found in the umbilical cord were higher than those present in maternal serum [21]. Der p 1 was already identified as a cysteine protease, a property that may facilitate this allergen to penetrate the barrier [44]. Furthermore, the presence of specific IgG can assist in the generation and transport of immune complexes [22, 45]. While we could not determine whether the detected allergens are in a free form or in the presence of immune complexes, we predict that the immune complexes formation is feasible and could be responsible for transferring a portion of the antigens present in the umbilical cord. However, individual differences that alter the permeability to macromolecules of the placenta (such as fibrin deposits that are commonly observed in all pregnancies) can also influence the concentrations of allergen transported, especially in its free form [46].

Similar to the umbilical cord, the transport of both allergens and their specific antibodies could be detected in colostrum samples. This discovery represents the first time that environmental allergens, as well as specific IgA and IgG, were found in human colostrum. The fact that we found a correlation between Der p 1 levels and Blo t 5 in colostrum indicates that some mothers are predisposed to carry higher concentrations of exogenous proteins in breast milk. The IgG in colostrum can be locally synthesized in the mammary gland [47] or from the maternal serum, based on the observation that intravenous administration of Ig resulted in immunodeficient mothers in the presence of IgG in human milk [48]. Although not shown, we can assume that there are two possible routes that allow proteins present in maternal serum to be transferred to the mammary gland: transcellular and paracellular routes. The first is dependent on the permeability of the mammary gland, whereas the second is dependent on the presence of immune complexes and can be mediated by the FcRn receptor expressed in epithelial cells of the mammary gland [49]. In accord with our findings, Vance and colleagues also detected a higher concentration of albumin in milk (0.12–1258 ng/ml) compared with paired umbilical cord samples (0.05–5.67 ng/mL) [43].

When comparing levels of specific IgG subclasses between Dp and Bt, specific IgG2 levels were higher for Bt than for Dp; by contrast, specific IgG3 levels were higher for Dp than for Bt. IgG2 is the dominant response to polysaccharide antigens, whereas IgG1 and IgG3 are the dominant responses to protein antigens [50]. Although there is a difference in reactivity between IgG2 and IgG3 against different antigens, we cannot state that it occurs because of the antigen structure because we used whole antigen extract in the assays.

Secretory IgA is the most important immunoglobulin in human milk, and the main function of IgA is to defend a child against enteric infections; however, there is evidence that IgA not only prevents allergic manifestations but also exerts immunoregulatory effects [51–54]. Specific IgA to both mites were detected in all samples analyzed, whereas specific IgG was detected in a high percentage of colostrum samples: 93.5% and 94.8% for IgG anti-Dp and anti-Bt, respectively (Table 4). The presence of IgA in the colostrum samples could be important to eliminate not only food allergens but also respiratory allergens because studies with animals have shown that once inhaled, most of allergens go to the gastrointestinal tract [55, 56]. Considering the immature immune system of the fetus, maternal antibodies are very important for neutralizing some pathogens, particularly early in life when children start interacting with many environmental antigens. Additionally, a review that analyzed 56 publications between 1966 and 2001 reported that over 50% of these studies showed that breast milk protects against atopic dermatitis and asthma, especially in children with a parental history of atopy [57].

In summary, we observed that Der p 1, Blo t 5, and specific antibodies to Dp and Bt are transferred to fetuses through the placenta and to newborns and infants via breastfeeding. Additionally, even with the low percentage of allergens found in the cord blood, specific IgM for both allergens could be detected in almost all samples collected, suggesting that it may have occurred sensitization of fetuses against Dp and Bt. The attempts to correlate allergic diseases with allergen exposure or even to protective effects in early infancy have not yielded conclusive results. Many aspects of allergen exposure still need to be clarified, and the fact that it may occur before birth indicates the need to investigate its relationship with the onset of allergies. Further studies are necessary to elucidate the role of these components during the development of the immune system and to determine how they eventually drive tolerance or allergies in children.

## Supporting Information

S1 Fig Correlations between specific IgG subclasses to Der p 1 and Blo t 5 in cord blood and mother sera. (PDF)

S2 Fig Correlations between IgM and Der p 1 or Blo t 5 in cord blood. (PDF)
