# Supplementary figures and images for: Early Exposure to Respiratory Allergens by Placental Transfer and Breastfeeding
Source: PLoS One. 2015 Sep 23;10(9):e0139064. doi: 10.1371/journal.pone.0139064 (PMC4580413; doi:10.1371/journal.pone.0139064)

Supplemental figure 1

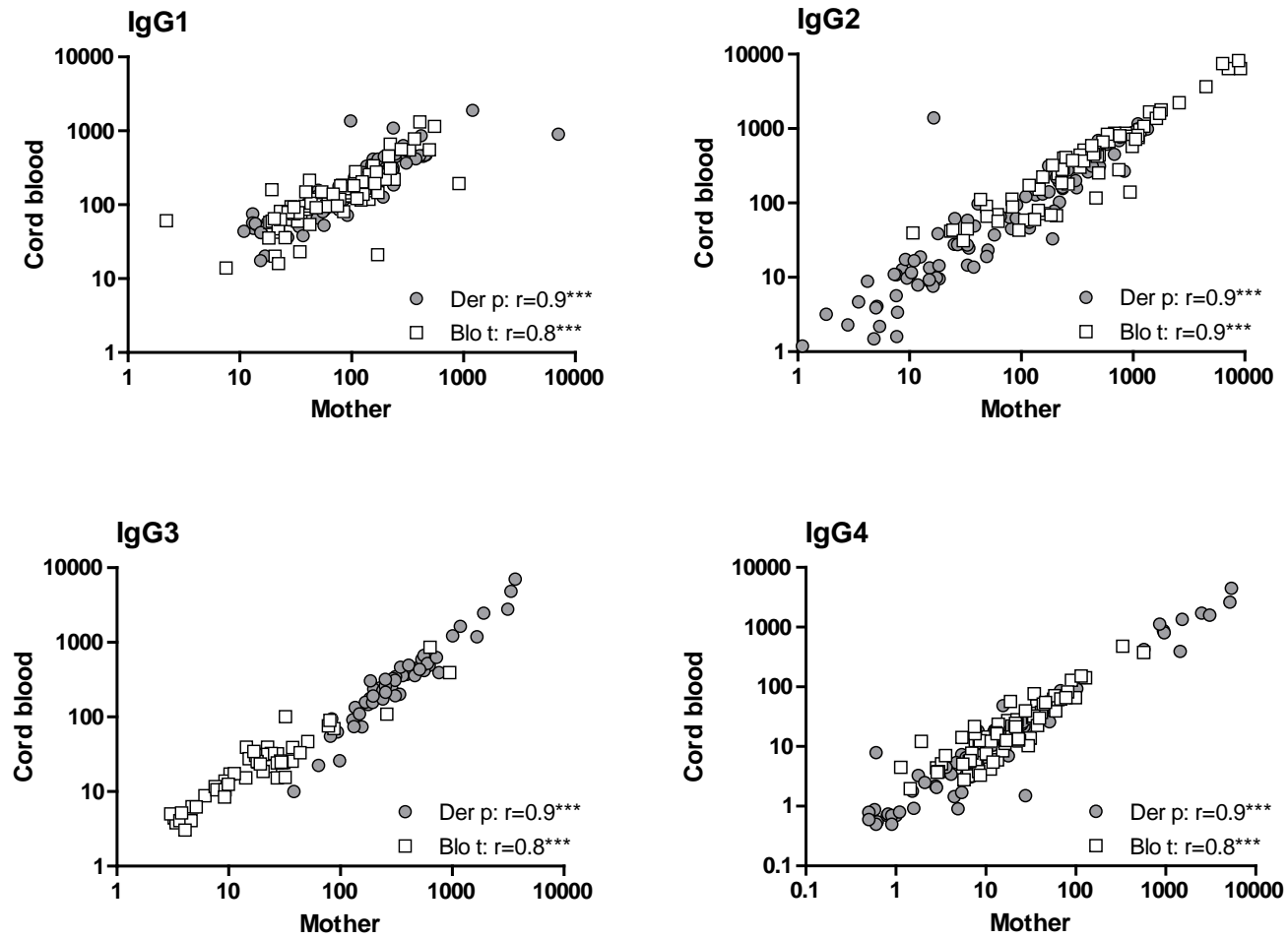

Supplement: S1 Fig — (PDF) [file pone.0139064.s001.pdf]

**Supplemental figure 2**

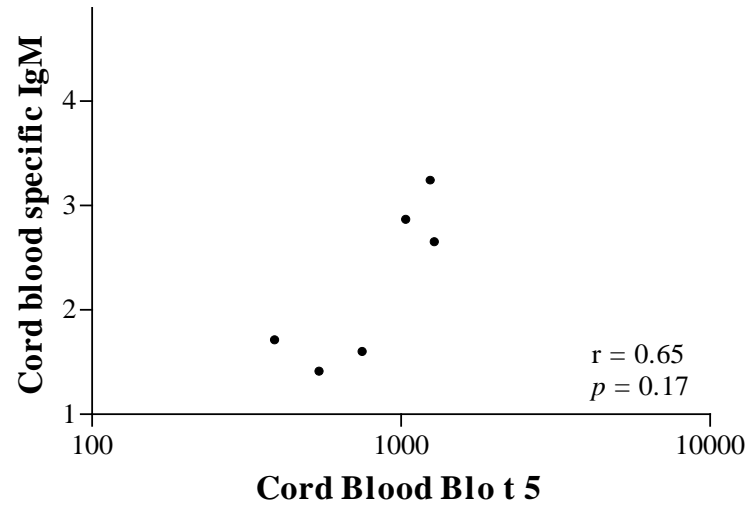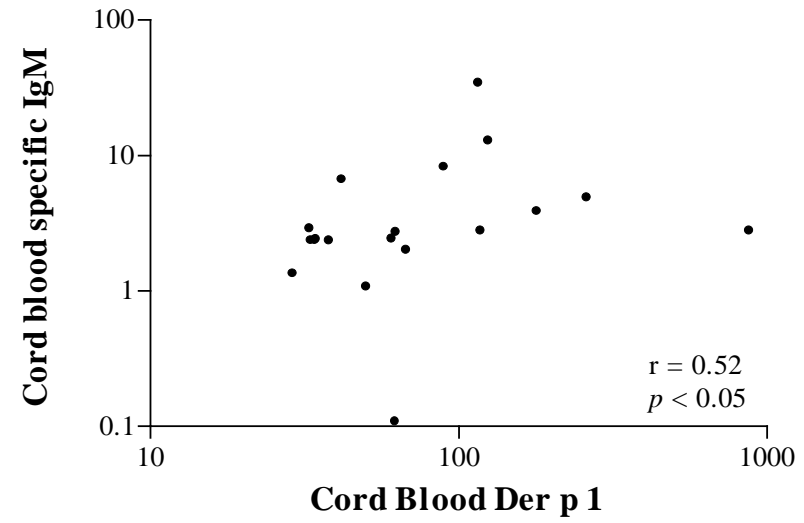

Supplement: S2 Fig — (PDF) [file pone.0139064.s002.pdf]
